# Prevalence and determinants of type 2 diabetes among lean African migrants and non-migrants: the RODAM study

**DOI:** 10.7189/jogh.09.020426

**Published:** 2019-12

**Authors:** Felix P Chilunga, Peter Henneman, Karlijn AC Meeks, Erik Beune, Ana Requena-Méndez, Liam Smeeth, Juliet Addo, Silver Bahendeka, Ina Danquah, Matthias B Schulze, Joachim Spranger, Ellis Owusu-Dabo, Kerstin Klipstein-Grobusch, Marcel MAM Mannens, Charles Agyemang

**Affiliations:** 1 Department of Public Health, Amsterdam Public Health Research Institute, Amsterdam UMC, University of Amsterdam, Amsterdam, the Netherlands; 2 Department of Clinical Genetics, Amsterdam UMC, University of Amsterdam, Amsterdam, the Netherlands; 3 Center for Research on Genomics and Global Health, National Human Genome Research Institute, National Institutes of Health, Bethesda, Maryland, USA; 4 ISGlobal, Barcelona Centre for International Health Research (CRESIB), Hospital Clinic, University of Barcelona, Barcelona, Spain; 5 Department of Non-communicable Disease Epidemiology, London School of Hygiene and Tropical Medicine, London, United Kingdom; 6 Department of Medicine, MKPGMS-Uganda Martyrs University, Kampala, Uganda; 7 Department of Molecular Epidemiology, German Institute of Human Nutrition, Nuthetal, Germany; 8 Institute for Social Medicine, Epidemiology and Health Economics, Berlin Institute of Health, University of Berlin, Berlin, Germany; 9 Clinic of Endocrinology, Diabetes and Metabolism, Berlin Institute of Health, University of Berlin, Berlin, Germany; 10 School of Public Health, Kwame Nkrumah University of Science and Technology, Kumasi, Ghana; 11 Julius Global Health, Julius Centre for Health Sciences and Primary Care, University Medical Centre Utrecht, Utrecht University, Utrecht, the Netherlands; 12 Division of Epidemiology and Biostatistics, School of Public Health, Faculty of Health Sciences, University of the Witwatersrand, Johannesburg, South Africa

## Abstract

**Background:**

Exposure to adverse conditions earlier in life-course can predispose to type 2 diabetes in adulthood, irrespective of body mass index (BMI). However, the burden of type 2 diabetes in lean Africans is not well understood despite higher exposure to adverse early life conditions. Mirroring ongoing epidemiological transition, we assessed the burden and determinants of type 2 diabetes in a homogenous group of lean Ghanaians residing in rural and urban Ghana, and as migrants in Europe.

**Methods:**

Baseline data from 2179 RODAM study participants with BMI<25kg/m^2^ (25-70 years) were analyzed. Prevalence and determinants of type 2 diabetes were estimated using logistic regression analysis. Adjustments were made for socio-demographic and lifestyle factors, use of anti-diabetic medication and optimal blood glucose control.

**Results:**

Prevalence of type 2 diabetes in rural, urban and migrant lean participants were 3.5%, 8.9% and 7.5% respectively, representing 55.4%, 35.6%, 13.2% of all participants with type 2 diabetes. Compared with lean rural participants, the odds of type 2 diabetes were higher in lean urban participants (adjusted OR = 8.81, 95% CI = 6.56-11.06), followed by migrants (5.27, 95% CI = 3.51-6.91). Irrespective of site, determinants of type 2 diabetes in lean participants include; presence of hypertension, physical inactivity, hypercholesterolemia and age (>45 years).

**Conclusions:**

Our study shows a high prevalence of type 2 diabetes among lean African populations in different geographical settings. Future studies are needed in-order to examine how contextual differences are related to the pathophysiology of type 2 diabetes in lean individuals.

It is well established that there is an emerging burden of type 2 diabetes and other cardio-metabolic diseases in sub-Saharan Africa (SSA) [1,2]. Although obesity is a well-known risk factor for type 2 diabetes, populations from low and middle income countries (LMIC) are at increased risk of type 2 diabetes at lower levels of body mass index (BMI) [3]. This is in contrast to high-income countries (HIC) where only about 20% of individuals with type 2 diabetes have normal BMI [4,5]. For many SSA countries, where health care resources are severely constrained, occurrence of type 2 diabetes in underweight/normal individuals poses major public health challenges such as increased mortality as a result of obesity paradox, increased rates of colorectal cancer and inapplicability of universally recommended lifestyle interventions such as body weight reduction [6-8].

Type 2 diabetes in underweight/normal weight individuals has been attributed to exposure to adverse conditions earlier in life-course [9,10]. This is in addition to the classical risk factors for type 2 diabetes such as alcohol consumption, smoking and physical inactivity [7]. Despite the higher rates of maternal undernutrition, pre-natal injurious agents and early childhood undernutrition in SSA, the burden of type 2 diabetes in underweight/normal weight Africans is still not well understood [11]. Additionally, there is need for diabetes control strategies to be applied according to specific rural, urban and migrant contexts due to rapid urbanization, migration and epidemiological transition [1]. However, little is also known about the determinants of type 2 diabetes in each of the rural, urban and migrant SSA contexts.

Current reports show that the major pathophysiology of type 2 diabetes in underweight/normal weight individuals is rapid beta cell failure as opposed to insulin resistance [8]. Therefore, investigating the burden of type 2 diabetes in non-obese populations and respective determinants in each geographical context will expand knowledge well beyond the connection between lifestyle factors, insulin resistance and type 2 diabetes. Novel aspects of the relationship between developmental origins of disease and pathophysiology of type 2 diabetes can be unraveled, thereby presenting opportunities for groundbreaking approaches to preventing and managing the disease [12].

In order to accentuate the environmental contributions to development of type 2 diabetes among underweight/normal weight individuals and mitigate the effects of dissimilar genetic background, it is imperative that a homogenous group of people is studied [13]. We, therefore, used data from a homogenous group of Ghanaians who participated in the Research on Obesity and Diabetes among African Migrants (RODAM) study in order to 1) assess the prevalence of type 2 diabetes in underweight/normal weight SSA populations in rural, urban and migrant contexts. 2) assess the proportion of types 2 diabetes cases comprised of underweight/normal weight populations in each SSA context. 3) assess the determinants of type 2 diabetes in underweight/normal weight populations in each SSA context. 4) assess the relative contribution of beta cell failure and insulin resistance to type 2 diabetes in underweight/normal weight SSA populations.

## METHODS

### Study setting and population

The multicentre RODAM study was initiated in 2012 with the aim of understanding the complex interplay between the environment and genetics in the development of obesity and diabetes among African migrants. The full details of the study have been published elsewhere [13]. In brief, 5898 Ghanaian men and women aged 25-70 years were recruited in Europe and in Ghana. In Europe, participants were recruited from the cities of Amsterdam (Netherlands), Berlin (Germany) and London (United Kingdom). In Ghana, recruitment of participants in the urban area was conducted in two purposively chosen cities (Kumasi and Obuasi), while recruitment in the rural area was conducted in 15 villages in the Ashanti region. A standardised approach for questionnaires, anthropometric measurements and venepuncture samples was used across all study sites. The response rates were 67% in Amsterdam, 68% in Berlin, 75% in London, 76% in rural Ghana and 74% in urban Ghana. Since our study was designed to study international migration, we categorized Ghanaians living in Europe as migrants while Ghanaians living in Ghana were considered as non-migrants.

### Ethical approval and consent to participate

Ethical approval was obtained from ethics committees of involved institutions in Ghana, Netherlands, Germany and UK before the start of data collection. All participants gave written informed consent.

### Underweight/normal weight individuals with Type 2 diabetes

Presence of type 2 diabetes was defined using the World Health Organization (WHO) diagnostic criteria (fasting blood glucose, ≥7.0 mmol/L, or current use of medication prescribed to treat type 2 diabetes or a previous self-reported diagnosis of type 2 diabetes a health professional. We used the current WHO definition of low/normal weight (ie, BMI<25kg/m^2^) to identify underweight/normal weight individuals in our study [14].

### Measurements

The following measurements were obtained through a structured questionnaire; age, sex, educational attainment, history of type 2 diabetes, use of medication for type 2 diabetes, use of dietary treatment for type 2 diabetes, physical activity levels, alcohol consumption, smoking and length of stay in Europe. Education was categorised as follows; (1) none or elementary, 2) lower secondary, 3) higher secondary, and 4) tertiary. Levels of physical activity were calculated using the Global Physical Activity Questionnaire (GPAQ) version 2, and categorised into a low, moderate or high levels based on the GPAQ criteria [15]. Alcohol consumption was categorised into no consumption, or any consumption. Smoking was categorised into current smokers, past smokers or non-smoker. Body weight was measured in light clothing and without shoes with SECA 877 scales to the nearest 0.1 kg. Participants height was measured without shoes with a portable stadiometer (SECA 217) to the nearest 0.1 cm. BMI was calculated as weight (kg) divided by height squared (m^2^). Central obesity was defined as waist-to-hip ratio (WHR) of ≥0.95 for men and ≥0.85 for women. Blood pressure (BP) was measured three times using appropriate cuffs in a sitting position after at least 5 minutes of rest (mmHg). The mean of the last two measurements was used in the analysis.

All biochemical analyses were performed in Berlin with an ABX Pentra 400 chemistry analyser (ABX Pentra; Horiba ABX, Montpellier, France). Concentration of total cholesterol, low density lipoprotein (LDL)-cholesterol, high density lipoprotein (HDL)-cholesterol and triglycerides was assessed using colorimetric test kits (mmol/L). Insulin concentrations were assessed using the Mercodia ELISA kit in pmol/L. Fasting plasma glucose concentration was measured using an enzymatic method (hexokinase) in mmol/L. Glycated hemoglobin (HbA1c) was measured in % and mmol/mol (HPLC). Hypercholesterolemia was defined as total cholesterol ≥200mg/dL, low density lipoprotein (LDL) -cholesterol ≥160mg/dL, high density lipoprotein (HDL)-cholesterol ≤40mg/dL, triglycerides ≥150mg/dL or medication with lipid lowering drugs. Impaired fasting glucose (IFG) was defined as a fasting glucose between 5.6 and 6.9 mmol/L, according to American Diabetes Association. Insulin sensitivity and beta cell function were assessed using Homeostatic Model Assessment (HOMA): HOMA-derived insulin resistance index (HOMA-IR) and HOMA-derived beta cell function (HOMA-B) [16]. HbA1c <7% (53 mmol/mol) was used to show optimal blood glucose control in individuals with type 2 diabetes for the preceding two to three months.

### Data analysis

Data analyses were performed using Stata 14 (Stata Corp LP, Texas 77845, USA). Summary statistics were presented as proportions for categorical variables and as means (with standard deviations) for normally distributed continuous variables or medians (with IQR-interquartile range) for skewed continuous variables. The differences of baseline characteristics in rural, urban and migrant participants were tested by Analysis of Variance (ANOVA) for normally distributed continuous variables, Kruskal-Wallis test for skewed continuous variables and χ^2^ tests for categorical variables. Prevalence of type 2 diabetes was calculated and standardised for age with a direct method. Logistic regression models were used to calculate odds ratios with corresponding 95% Confidence Intervals (CI) for type 2 diabetes in underweight/normal weight participants by location of current residency (rural Ghana as the reference category), adjusting for socio-demographic factors. In multivariable models, adjustments were made for alcohol consumption, smoking, physical activity, blood pressure, receiving treatment for type 2 diabetes (weight-loss diet, oral medications and insulin use), level of blood glucose control (HbA1c <7% /53mmol/mol) and length of stay for migrants. For adjustment variables, missing values represented <5% of the data in every variable. In addition, univariate and multivariate logistic regression models were used to assess the associations between type 2 diabetes in underweight/normal weight individuals and its determinants by location of current residency.

In a sub-set of underweight/normal weight participants with IFG, we assessed the association of HOMA-IR and HOMA-B with IFG using logistic regression models. This sub-analysis was carried out in order to identify whether beta cell failure or insulin resistance was more associated with the development of type 2 diabetes in underweight/normal weight individuals. In these models; first, we calculated the inverse of HOMA-B (ie, 1/HOMA-B). Second, we converted both HOMA-IR and inverse HOMA-B into standardized z scores in order to achieve comparability of the variables. Third, we calculated the odds of IFG by 1SD increase in HOMA-IR and inverse HOMA-B adjusting for age, sex and location of residency. Lastly, we calculated the attributable risk for inverse HOMA-B and HOMA-IR relative to IFG.

## RESULTS

### Baseline characteristics

A total of 2179 (37.1%) of all RODAM study participants were underweight/normal weight, of which, 853(39.1%) were rural residents, 583(26.8%) were urban residents and 743(34.1%) were migrants (Figure S1 in **Online Supplementary Document**). In these underweight/normal weight participants, the majority of migrants were male (61.2%), while the majority of rural and urban participants were females (53.6% and 55.6%, respectively). About half (50.1%) of underweight/normal weight participants were older than 45 years. Median age was highest in rural residents, who were also the least educated. Median length of stay in Europe among migrants was 13 years (IQR, 5.1-22.2). Rural participants were more likely to be former smokers, and had the highest levels of physical activity and triglycerides. Urban participants had the highest level of total cholesterol, LDL-cholesterol, hypercholesterolemia and WHR. Migrants had the highest level of mean BMI, HDL-cholesterol, blood pressure, and were more likely than rural and urban participants to drink alcohol and smoke (**Table 1**).

**Table 1:** Baseline characteristics of participants in migrant, urban and rural residents*

	Total (n = 2179)	Migrants (n = 743)	Urban residents (n = 583)	Rural residents (n = 853)
**Demographics**				
Women, n (%)	1069 (49.1)	288 (38.8)	324 (55.6)	457 (53.6)†
Mean age, years	45.07 (13.89)	41.17 (12.74)	44.59 (12.59)	48.77 (14.69)†
Age group, n (%):				
25-44	1070 (49.11)	429 (57.74)	290 (49.74)	351 (41.15)^†^
45-65	898 (41.21)	287 (38.63)	257 (44.08)	354 (41.50)
>65	211 (9.68)	27 (3.63)	36 (6.17)	148 (17.35)
Education, n (%):				
Elementary	825 (40.52)	115 (17.04)	239 (42.45)	471 (59.02)^†^
Lower secondary	700 (34.38)	157 (34.67)	227 (40.32)	239 (29.95)
Higher secondary	309 (15.18)	104 (26.81)	66 (11.72)	62 (7.77)
Tertiary	202 (9.92)	100 (21.48)	31 (5.51)	26 (3.26)
**Lifestyle risk factors**				
BMI, n (%):				
<18	206 (9.45)	14 (1.88)	33 (5.66)	159 (18.64)†
18.0-24.9	1973 (90.55)	729 (98.12)	550 (94.34)	694 (81.36)
Median BMI, kg/m^2^	22.01 (20.24-23.68)	23.20 (21.69-24.16)	22.08 (20.64-23.73)	20.58 (18.97-22.34)†
Raised waist-to-hip ratio (WHR), n (%)	1074 (49.36)	279 (37.65)	330 (56.70)	465 (54.51)
Mean total cholesterol, mmol/L	4.67 (1.12)	4.85 (1.08)	4.94 (1.08)	4.35 (1.10)c
Mean HDL cholesterol, mmol/L	1.32 (0.41)	1.48 (0.39)	1.29 (0.33)	1.20 (0.38)†
Mean LDL cholesterol, mmol/L	2.91 (0.94)	2.99 (0.94)	3.18 (0.92)	2.67 (0.91)†
Mean Triglycerides, mmol/L	0.85 (0.65-1.14)	0.71 (0.56-0.94)	0.89 (0.69-1.19)	0.95 (0.73-1.23)†
Hypercholesterolemia, n (%)	83 (5.94)	13 (2.24)	41 (11.06)	29 (6.79)
Mean Systolic BP, mmHg	125.52 (19.91)	128.40 (17.34)	124.46 (21.45)	123.78 (20.65)†
Mean Diastolic BP, mmHg	78.22 (12.04)	80.77 (11.63)	77.60 (12.59)	76.41 (11.63)†
Hypertension, n (%)	609 (27.95)	257 (34.59)	142 (24.36)	210 (24.62)†
High level physical activity, n (%)	1023 (54.18)	254 (47.65)	288 (51.43)	481 (60.50)†
Smoking, n (%):				
Current	87(4.28)	54(8.00)	10(1.77)	23(2.89)^‡^
Past	156(7.67)	49(7.26)	39(6.91)	68(8.54)^†^
Any alcohol consumption, n (%)	853 (39.15)	320 (43.07)	169 (28.99)	364 (42.67)^†^
Median length of stay, years	13.12 (5.11-22.21)	13.12 (5.11-22.21)		
**Diabetes related**				
Median fasting blood glucose, mmol/L	4.95 (4.60-5.32)	4.91 (4.57-5.33)	5.04 (4.71-5.40)	4.89 (4.55-5.27)^†^
Median Insulin levels,(pmol/L)	3.9 (2.5-6.1)	4.3 (2.8-6.3)	4.4 (2.4-6.8)	3.4 (2.2-5.3)^†^
Impaired Fasting Glucose, n (%)	217 (10.58)	80 (11.48)	57 (10.71)	80 (9.73)^†^
Mean HOMA-IR	1.19 (1.53)	1.24 (1.92)	1.35 (1.48)	1.01 (1.09)^†^
Mean Inverse HOMA-B	0.03 (0.05)	0.02 (0.03)	0.03 (0.08)	0.02 (0.04)^†^
Diabetics, n (%)	128 (5.87)	46 (6.19)	51 (8.75)	31 (3.63)^†^
On diabetes treatment, n (%)	60 (46.87)	27 (58.69)	25 (49.02)	8 (25.81)^‡^
On diabetic diet, n (%)	37 (61.67)	11 (40.74)	18 (72.0)	8 (100.0)
On oral anti-diabetic drugs, n (%)	54 (90.0)	21 (77.78)	25 (100.0)	8 (100.0)
On Insulin treatment, n (%)	3 (5.0)	4 (14.81)	3 (12.0)	0
HbA1c levels <7% (53 mmol/mol), n (%)	28 (46.67)	17 (62.96)	7 (28.00)	4 (50.00)

IQR – interquartile range; SD – standard deviation, BMI – body mass index, HDL – high density lipoprotein, LDL – low density lipoprotein, BP – blood pressure, HOMA – homeostatic model assessment, HOMA-IR – HOMA-derived insulin resistance index (HOMA-IR), and HOMA-derived beta cell function (HOMA-B), HbA1c – glycated hemoglobin

*Data are n (%), median (interquartile range, IQR), or mean (standard deviation, SD).

†*P* for difference in baseline characteristics between migrants, urban and rural participants <0.001.

‡*P* for difference in baseline characteristics between migrants, urban and rural participants >0.05.

### Type 2 diabetes in underweight/normal weight individuals

Median fasting blood glucose and insulin levels were highest in underweight/normal weight urban residents. The crude prevalence of type 2 diabetes in underweight/normal weight participants was 3.6% among rural residents, 8.8% among urban residents and 6.2% among migrants, with an overall prevalence of 5.9%. About 47% of all underweight/normal weight individuals with type 2 diabetes were on treatment (diet and medication). The most common mode of treatment was oral-antidiabetic agents in all three groups. Use of insulin for type 2 diabetes treatment was more common in migrants. The majority (63%) of underweight/normal weight migrants on type 2 diabetes treatment had achieved optimal blood glucose control in the preceding 2-3 months (**Table 1**).

**Figure 1:**
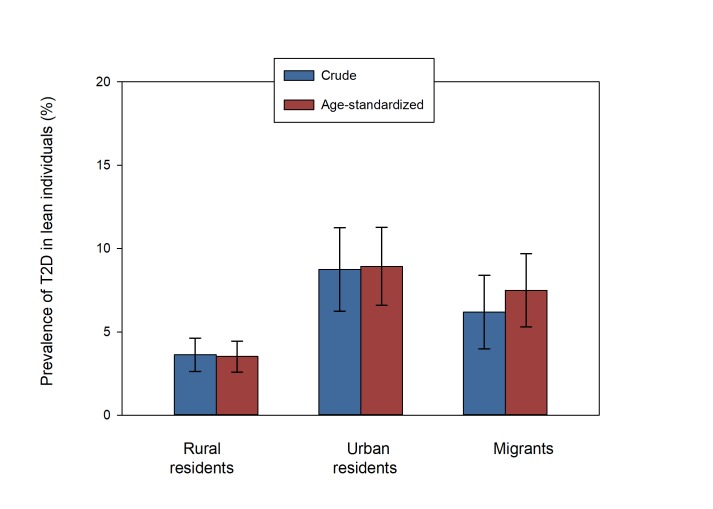
Crude and age -standardized prevalence of type 2 diabetes in underweight/normal weight urban residents, rural residents and migrants. Age-standardized prevalence of type 2 diabetes in underweight/ normal weight participants includes confidence intervals as follows; rural residents 3.52% (95% CI = 2.29-4.45), urban residents 8.93% (95% CI = 6.59-11.27), migrants 7.49% (95% CI = 4.89-6.85). T2D – type 2 diabetes mellitus.

**Figure 2:**
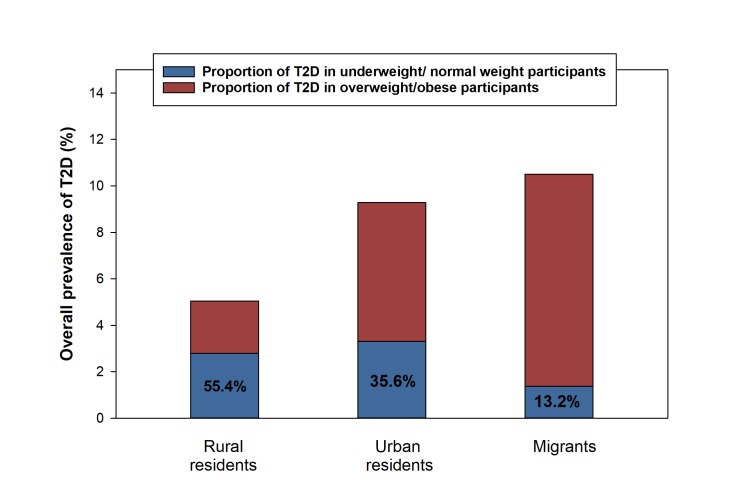
Proportion of type 2 diabetes in underweight / normal weight participants within the RODAM study. T2D – type 2 diabetes mellitus.

**Figure 3:**
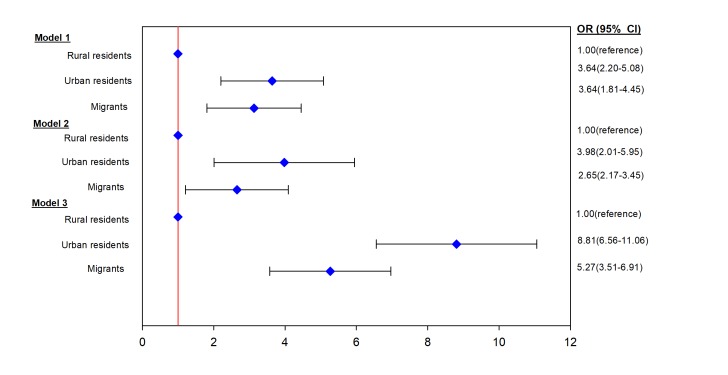
Odds of type 2 diabetes in underweight/normal weight urban residents and migrants, compared to rural – Ghanaians. Data are odds ratio (95% Confidence Interval). Model 1 – adjusted for age, sex and education. Model 2 – Model 1 with further adjustment for alcohol consumption, smoking, physical activity, hypercholesterolemia, hypertension and length of stay in the migrants. Model 3 – Model 2 with further adjustment for blood glucose control (HbA1c <7% (53 mmol/mol)) and diabetes treatment (diet, oral medication and insulin use).

When standardized for age, prevalence of type 2 diabetes in underweight/normal weight individuals was 3.5%, 8.9% and 7.5% in rural residents, urban residents, and migrants, respectively (**Figure 1**). The proportions of type 2 diabetes in underweight/normal weight individuals relative to all type 2 diabetes cases were 55.4% for rural residents, 35.6% for urban residents and 13.2% for migrants (**Figure 2**). Compared to rural residents, the odds of type 2 diabetes in underweight/normal weight individuals were higher in both urban residents (OR = 8.81, 95% CI = 6.56-11.06) and migrants (OR = 5.27, 95% CI = 3.51-6.91) compared to rural residents when adjusted for other factors (**Figure 3**).

In the subset of 217 underweight/normal weight individuals with IFG (compared to those with normal blood glucose), the odds for IFG were higher per 1SD increase in inverse HOMA-B (AOR = 16.07, 95% CI = 10.16-25.41) compared to HOMA-IR (AOR = 9.83, 95% CI = 6.91-13.99), with correspondingly higher attributable risk (AR)% for inverse HOMA-B (AR% 43.57, 95% CI = 35.30-51.84) than for HOMA-IR (AR% 32.86, 95% CI = 27.02-38.70) (Table S1 in **Online Supplementary Document**).

### Determinants of type 2 diabetes in underweight/normal weight participants

In underweight/normal weight rural residents, presence of hypertension (AOR = 2.24, 95% CI = 1.82-6.09) and high levels of physical activity (AOR = 0.22, 95% CI = 0.06-0.78) were independent determinants of type 2 diabetes. In underweight/normal weight urban residents, middle age (45-65 years) (AOR = 4.89, 95% CI = 1.66-14.40), hypercholesterolemia (AOR = 10.53, 95% CI = 3.85-28.79) and presence of hypertension (AOR = 3.76, 95% CI = 1.62-8.72) were independent determinants of type 2 diabetes. In underweight/normal weight migrants, presence of hypertension (AOR = 4.45, 95% CI = 1.38-14.32) and older age (>65 years) (AOR = 9.84, 95% CI = 2.22-23.71) were independently associated with type 2 diabetes (**Table 2**).

**Table 2:** Logistic association between socio-demographic and lifestyle risk factors with type 2 diabetes in underweight/normal weight migrant, urban and rural participants*

	All participants, N = 2179		Migrants, OR (95% CI), n = 743		Urban Residents, OR (95% CI), n = 583		Rural residents, OR (95% CI), n = 853	
**Univariate analysis**	**Multivariate analysis†**	**Univariate analysis**	**Multivariate analysis†**	**Univariate analysis**	Multivariate analysis*	**Univariate analysis**	Multivariate analysis*
**Sex:**								
Male	1.00 (ref)	1.00 (ref)	1.00 (ref)	1.00 (ref)	1.00 (ref)	1.00 (ref)	1.00 (ref)	1.00 (ref)
female	0.84 (0.57-1.24)	0.8 (0.47-1.45)	0.42 (0.19-0.91)	0.29 (0.08-1.21)	0.79 (0.19-0.91)	0.77 (0.31-1.29)	1.98 (0.85-4.62)	2.01 (0.54-7.53)
**Age group:**								
18-44	1.00 (ref)	1.00 (ref)	1.00 (ref)	1.00 (ref)	1.00 (ref)	1.00 (ref)	1.00 (ref)	1.00 (ref)
45-65	3.57 (2.29 -5.57)	2.28 (1.31-3.98)	2.46 (1.24-4.88)	1.79 (0.69-4.69)	3.65 (1.79-4.35)	2.78 (1.16-6.65)	5.94 (1.99-17.72)	2.15 (0.61-7.68)
>65	3.31 (1.73 -6.32)	2.62 (1.04-4.99)	10.86 (3.52-33.44)	9.84 (2.22-23.71)	4.89 (1.66-14.40)	2.69 (0.71-10.23)	2.56 (0.62-10.72)	0.77 (0.13-4.51)
**Education:**								
Elementary	1.00 (ref)	1.00 (ref)	1.00 (ref)	1.00 (ref)	1.00 (ref)	1.00 (ref)	1.00 (ref)	1.00 (ref)
Lower secondary	1.33 (0.85-2.07)	1.50 (0.81-2.79)	0.78 (0.33-1.87)	1.41 (0.28-7.06)	1.32 (1.79-7.45)	1.60 (0.68-3.74)	0.95 (0.38-2.42)	1.39 (0.37-5.28)
Higher secondary	1.30 (0.77-2.41)	1.09 (0.47-2.51)	0.81 (0.32-2.01)	1.41 (0.26-7.78)	0.56 (0.15-2.05)	0.29 (0.04-2.48)	3.04 (0.89-10.38)	3.15 (0.69-14.89)
Tertiary	1.23 (0.61-2.52)	0.97 (0.37-2.61)	0.52 (0.18-1.53)	0.86 (0.15-5.19)	1.10 (0.29-4.27)	1.08 (0.21-5.61)	2.01 (0.24-16.85)	1.19 (0.33-30.97)
**Hypercholesterolemia:**								
No	1.00 (ref)	1.00 (ref)	1.00 (ref)	1.00 (ref)	1.00 (ref)	1.00 (ref)	1.00 (ref)	1.00 (ref)
Yes	4.54 (2.42-8.53)	5.66 (2.86-11.23)	1.99 (1.36-11.03)	2.84 (0.29-27.21)	13.31 (5.08-34.88)	10.53 (3.85-28.79)	3.99 (1.34-11.94)	2.99 (0.84-10.71)
**Hypertension:**								
No	1.00 (ref)	1.00 (ref)	1.00 (ref)	1.00 (ref)	1.00 (ref)	1.00 (ref)	1.00 (ref)	1.00 (ref)
Yes	3.49 (2.35-5.19)	3.94 (2.19-7.07)	3.36 (1.66-6.81)	4.45 (1.38-14.32)	3.81 (1.98-7.32)	3.76 (1.62-8.72)	2.79 (1.26-6.22)	2.24 (1.82-6.09)
**Physical activity:**								
Low level	1.00 (ref)	1.00 (ref)	1.00 (ref)	1.00 (ref)	1.00 (ref)	1.00 (ref)	1.00 (ref)	1.00 (ref)
Medium level	0.79 (0.48-1.32)	0.88 (0.44-1.76)	1.08 (0.31-3.83)	1.11 (0.37-3.31)	1.19 (0.52-2.74)	1.01 (0.31-3.35)	0.47 (0.18-1.24)	0.59 (0.15-2.28)
High level	0.46 (0.29-0.73)	0.59 (0.33-1.06)	0.69 (0.24-2.03)	0.78 (0.30-2.03)	0.68 (0.38 -1.39)	0.83 (0.34-2.05)	0.19 (0.08 -0.51)	0.22 (0.06-0.78)
**Smoking:**								
Never	1.00 (ref)	1.00 (ref)	1.00 (ref)	1.00 (ref)	1.00 (ref)	1.00 (ref)	1.00 (ref)	1.00 (ref)
Current	1.64 (0.58-4.66)	5.76 (0.74-47.81)	1.42 (0.46-4.31)	3.80 (0.46-6.50)	1.00	1.00	1.00	1.00
Past	1.83 (0.58-5.79)	4.66 (0.52-43.23)	1.17 (0.26-5.18)	4.09 (0.32-5.28)	0.76 (0.27-2.08)	1.01 (0.25-3.96)	1.54 (0.79-3.11)	2.11 (0.21-21.04)
**Alcohol consumption:**								
None	1.00 (ref)	1.00 (ref)	1.00 (ref)	1.00 (ref)	1.00 (ref)	1.00 (ref)	1.00 (ref)	1.00 (ref)
Any	1.01 (0.69-1.49)	1.32 (0.78-2.25)	1.28 (0.69-2.39)	2.05 (0.78-5.32)	1.17 (0.61-2.25)	1.18 (0.58-2.44)	0.88 (0.39-1.99)	1.22 (0.52-2.33)

OR – odds ratio, CI – confidence interval

*Data are odds ratio (95% confidence intervals).

†Multivariate analysis – adjusted for demographic factors such as age, sex, education, alcohol consumption, smoking, physical activity, hypercholesterolemia; hypertension, blood glucose control (HbA1c <7% (53 mmol/mol)), diabetes treatment (dieting, oral medication and insulin use) accordingly.

## DISCUSSION

### Key findings

Our study shows that type 2 diabetes in underweight/normal weight individuals is very common in different Ghanaian contexts. The highest prevalence of type 2 diabetes in all underweight/normal weight individuals is in urban residents. However, underweight/normal weight individuals with type 2 diabetes make up the highest proportion of all type 2 diabetes cases in rural residents. Determinants of type 2 diabetes in underweight/normal weight individuals include; presence of hypertension and physical inactivity among rural residents, middle age (45-65 years), hypercholesterolemia and presence of hypertension among urban residents, older age (>65 years) and presence of hypertension among migrants.

### Interpretation of key findings

In our study, we found that the overall prevalence of type 2 diabetes in underweight/normal weight individuals was 5.9%, which is lower than the prevalence of type 2 diabetes in the overweight (10.3%) and obese (12.2%) reported from the same RODAM study [13]. This finding was expected since obesity is a well-known risk factor for type 2 diabetes, with the prevalence of type 2 diabetes increasing with increasing BMI [17]. In addition, our study found that the overall proportion of type 2 diabetes in underweight/normal weight individuals with respect to all type 2 diabetes cases was 24%, a figure which is higher than those reported in European or USA cohorts ie, 7.5% in the German DiaRegis (Diabetes treatment patterns and goal achievement in primary diabetes care) cohort, 8.4% in the German DIVE (Diabetes Versorgungs-Evaluation) cohort, 12% in the NHS (Nurses’ Health Study) and 16.5% in HPFS (Health Professionals Follow-Up Study) cohort [18-20]. This finding is expected considering that risk factors for type 2 diabetes in underweight/normal weight individuals such as maternal undernutrition, low birth weight, childhood undernutrition are more common in SSA populations, hence predisposing SSA populations (including international migrants living in Europe) to type 2 diabetes as opposed to their European host populations [11,21].

Our study is the first to mirror the epidemiological transition in Africa and report the differences in type 2 diabetes in underweight/normal weight individuals by location of residency. The highest odds of type 2 diabetes in underweight/normal weight individuals were higher in urban residents, followed by migrants. This finding was unexpected considering that fetal exposure to maternal undernutrition, pre-natal injurious agents and early childhood undernutrition are more common in the rural areas and could greatly increase the risk of type 2 diabetes in the rural group [16]. However, it should be noted that type 2 diabetes in underweight/normal weight rural residents accounted for the majority (55%) of all type 2 diabetes cases, with only 13% of all type 2 diabetes cases in migrants consisting of underweight/ normal weight individuals. In our study, differences in the measured risk factors did not explain the observed higher odds of type 2 diabetes in urban residents compared to migrants. Previous studies have shown that metabolically unhealthy normal weight (MUNW) individuals are at risk of type 2 diabetes than metabolically healthy normal weight (MHNW) individuals [7]. MUNW encompasses glucose elevation in the presence of impaired insulin secretion, hypercholesterolemia, low leg fat mass, visceral obesity and fatty liver [7]. It is likely that the unmeasured MUNW contributes to the higher odds of type 2 diabetes in urban residents compared to migrants, but this need to be explored with further studies.

Variation of type 2 diabetes determinants by geographical context in underweight/normal weight individuals was anticipated. First, previous studies have reported that underweight/normal weight individuals at risk of type 2 diabetes present with a lipodystrophy like syndrome [7]. Evidence from our study showed that underweight/normal weight urban residents had a greater proportion of hypercholesterolemia compared to other groups, which would increase the risk of type 2 diabetes in this group. However, hypercholesterolemia results from a complex interplay of diet, physical activity and genetics. The higher levels of hypercholesterolemia in the urban residents could also be a direct result of physical inactivity and unhealthy diets as observed in our study [22].

Second, there is substantial overlap between diabetes and hypertension, reflecting a significant overlap in their etiology and disease mechanisms, including the sympathetic nervous system, renin–angiotensin–aldosterone system (RAAS), oxidative stress, adipokines, and peroxisome proliferator-activated receptors (PPARs) [23]. Hypertension has been reported in about 50% to 80% of individuals with type 2 diabetes [24]. It has also been reported that hypertensive subjects are 2.5 times more likely to develop type 2 diabetes [25]. In our study, hypertension was a determinant of type 2 diabetes in underweight/normal weight individuals in all three study groups. However, the odds of type 2 diabetes from hypertension were highest in underweight/normal weight individual migrants who also had the highest levels of hypertension at baseline compared to other groups.

It is worth noting that, individuals diagnosed with type 2 diabetes at older ages (60 years in men and 70 years in women in SSA) tend to be less overweight due to loss of muscle mass, impairment of pancreatic function, and increased intra-abdominal fat [26,27]. Therefore, it was not surprising that old age was associated with type 2 diabetes in underweight/normal weight individuals, especially in migrants. Nevertheless, the effects of age on type 2 diabetes were not apparent in underweight/normal weight rural residents, even though this group had the highest number of old participants. Thus, it is unclear why old age is not associated with type 2 diabetes in the underweight/normal weight rural group.

It is well established that physical exercise is protective against type 2 diabetes [28]. In our study, underweight/normal weight individuals with type 2 diabetes were 50% less likely to have higher levels of physical activity compared to those without type 2 diabetes. High levels of physical activity were seen in rural residents, which may contribute to the lowest risk of type 2 diabetes in this group.

Alcohol consumption and smoking were not associated with type 2 diabetes in underweight/normal weight individuals. In contrast, previous studies have shown that alcohol consumption and smoking predispose individuals to type 2 diabetes in underweight/normal weight individuals, especially in the western context [7]. The lack of a significant association between alcohol consumption and smoking with type 2 diabetes in underweight/normal weight individuals in the current study may be due to the low prevalence of smoking and alcohol consumption among our study population. For example, the prevalence of smoking and high alcohol consumption were around 44.6% and 12% in the German DIVE study respectively,[18] whereas only 4.3% our respondents were smokers and 6.2% had any consumption of alcohol. It could be that our study did not have sufficient power to detect such associations.

The pathogenic mechanism of type 2 diabetes in underweight/normal weight individuals populations has been reported as predominantly beta cell failure, as opposed to insulin resistance, which is the major pathophysiology of type 2 diabetes in overweight/obese individuals [10]. In the subset of participants with IFG, we found higher odds of HOMA-B compared to HOMA-IR per increase in each SD. The attributable risk for HOMA-B was also higher than that of HOMA-IR. This finding shows that pancreatic beta cell failure also makes a relatively larger contribution to the pathophysiology of type 2 diabetes in underweight/normal weight SSA populations as opposed to insulin resistance [29].

### Strengths and limitations

The strength of our study is that it assesses a homogenous population of migrant and non-migrant Ghanaians living in different settings in Africa and Europe using standardised methods, which mitigates the effects the effects of studying different SSA populations with dissimilar genetic background. Several limitations of this study should be considered. First, our data are cross-sectional and rely on self-reported measures for socio-demographic and lifestyle risk factors hence we cannot preclude the possibility of recall bias. Second, some participants were underweight/normal weight due to weight loss effects of type 2 diabetes medication (biguanides, GLP-1 receptor agonists, SLGT2 inhibitors), or even poor control of blood glucose [30-32].We adequately controlled for these effects in the logistic regression models, thereby increasing the accuracy of our results. Third, we did not have data on early life adverse factors such as maternal undernutrition, low birthweight, prematurity, childhood malnutrition, epigenetics and measures of MUNW. Lastly, our numbers were too few to stratify the analysis by sex, hence we could not reported sex differences in our study.

## CONCLUSIONS

Our findings are highly relevant to SSA countries with a predominantly rural population and undergoing rapid urbanization, yet have limited resources to tackle type 2 diabetes. Underweight/normal weight individuals are neglected in the prevention of type 2 diabetes since current recommendations for screening and lifestyle modifications are advised at a BMI greater than 25kg/m^2^. Worse still, rural areas have limited capacity to manage the detrimental effects of type 2 diabetes due to constrained resources. From our results, it is apparent that future studies should examine the pathophysiology of type 2 diabetes in underweight/normal weight individuals, including associations with maternal undernutrition, low birth weight, prematurity, childhood malnutrition, epigenetics, and MUNW concept. Furthermore, type 2 diabetes control efforts in SSA and African populations in Europe should be extended to underweight/normal weight individuals taking into account differences in rural, urban and migrant contexts.

## Additional material

Online Supplementary Document
