# Supplementary material for: Prevalence and determinants of type 2 diabetes among lean African migrants and non-migrants: the RODAM study
Source: J Glob Health. 2019 Oct 22;9(2):020426. doi: 10.7189/jogh.09.020426 (PMC6815658; doi:10.7189/jogh.09.020426)
Supplement: Online Supplementary Document [file jogh-09-020426-s001.pdf]

**Figure S1: Flow chart of participation by site**

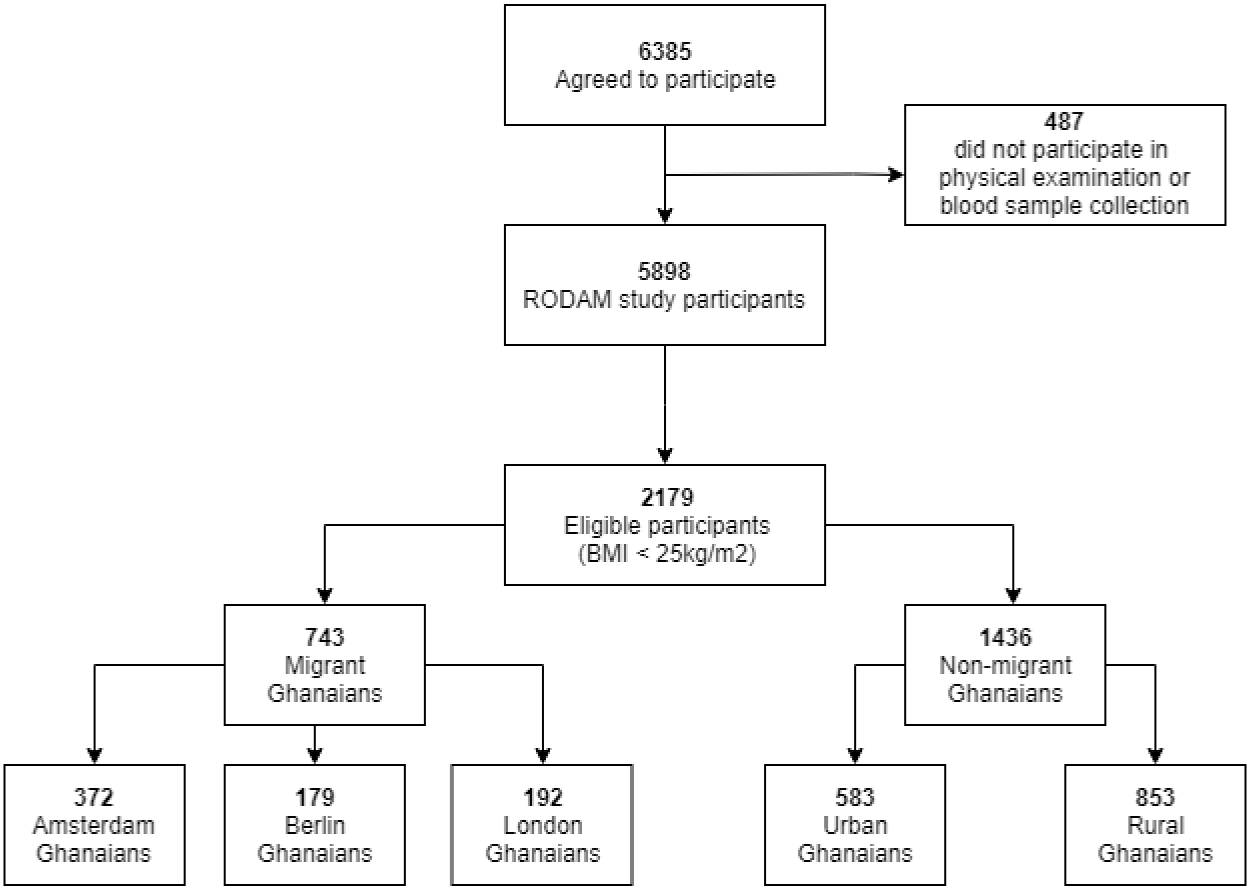

**Table S1: Adjusted odds ratios and attributable risk for impaired fasting glucose per SD increase in HOMA-IR AND Inverse HOMA-B**

| | | **Model 1*** | **Model 2 †** | **Attributable risk (AR) % for Model 2** |
|---|---|---|---|---|
| | **1SD** | OR (95%CI) | OR(95%CI) | AR(95%CI) |
| HOMA-IR‡ | 1.53 | 9.89(6.96-14.07) | 9.83(6.91-13.99) | 32.86(27.02-38.70) |
| Inverse HOMA-B‡ | 0.05 | 15.04(9.57-23.63) | 16.07(10.16-25.41) | 43.57(35.30-51.84) |

* Adjusted for age and sex

† Adjusted for age sex and location of residency (rural Ghana, urban Ghana and Europe)

‡ HOMA –IR and inverse HOMA-B were z-standardized to make them comparable
